# Advances in Drugs Targeting Lymphangiogenesis for Preventing Tumor Progression and Metastasis

**DOI:** 10.3389/fonc.2021.783309

**Published:** 2022-01-06

**Authors:** Chuqi Wang, Ming Chu

**Affiliations:** ^1^ Department of Immunology, School of Basic Medical Sciences, Peking University, National Health Commission (NHC) Key Laboratory of Medical Immunology (Peking University), Beijing, China; ^2^ I.M. Sechenov First Moscow State Medical University, Moscow, Russia

**Keywords:** lymphangiogenesis, VEGFR-2/-3, VEGF-C/-D, metastasis, tyrosine kinase inhibitor, angiogenesis, HGF, antibody

## Abstract

Metastasis of cancer cells from the primary tumor to other organs and tissues in the body is the leading cause of death in patients with malignancies. One of the principal ways cancer cells travel is through lymphatic vessels, and tumor invasion into the regional lymph nodes is a hallmark of early metastasis; thus, the formation of especially peritumoral lymphatic vessels is essential for tumor transportation that gives rise to further progression. In the past few decades, tumor-induced lymphangiogenesis has been testified to its tight correlation with lymphatic metastasis and poor clinical outcomes in multiple types of human malignancies, which warrants novel potential therapeutic targets for cancer treatment. As the understanding of underlying molecular mechanisms has grown tremendously over the years, an inexorable march of anti-lymphangiogenic therapy also aroused terrific interest. As a result, a great number of drugs have entered clinical trials, and some of them exhibited predominant contributions in cancer management. Herein, this review provides an updated summary of the current advances in therapies preventing lymphatic metastasis and discusses the validity of different applications.

## Introduction

Lymphangiogenesis is known as the formation of lymphatic vessels from pre-existing lymphatic vasculature. It participates in various physiological settings like homeostasis, immunity, embryonic development, and wound healing, while pathologically, this process is ordinarily implicated in organ graft rejection, lymphedema, and cancer metastasis. Additionally, other than the conventional views, many novel functions and roles of the lymphatic vasculature were uncovered more recently. Research has shown that an alteration of lymphangiogenesis can result in human pathologies such as obesity, aging, cardiovascular diseases including myocardial infarction and atherosclerosis, ocular diseases especially glaucoma, and inflammatory bowel diseases like Chron’s disease. Some neurological disorders, including neurodegenerative diseases like Alzheimer’s disease and Parkinson’s disease, and multiple sclerosis, can also be regulated by meningeal lymphatics (1). The correlation with cancer metastasis, whereas not equally solid in every tissue, occurred commonly in melanoma, breast cancer, colorectal cancer, and squamous cell carcinoma of head and neck (2). However, unlike tumor-induced angiogenesis which has been well recognized since decades ago and the first drug to inhibit this process, Avastin, became available in the clinics from 2004 (3). The initial interest in lymphangiogenesis started with the detection of pro-lymphangiogenic factors VEGF-C in 1996 (4) and VEGF-D in 1997 (5), causing relatively delayed development of anti-lymphangiogenic therapy compared to that of anti-angiogenic therapy. Fortunately, over the past few years, our growing understanding of the signaling events regulating lymphangiogenesis has advanced on the heels of investigations utilizing podoplanin, LYVE-1, PROX-1, desmoplakin, and VEGFR-3 as the lymphatic endothelial cell(LEC)-specific markers (6).

Tumor cells and tumor-associated inflammatory cells express lymphangiogenic growth factors as well as cytokines that initiate signaling cascades to drive lymphatic vessel growth *via* different LEC surface receptors. Despite the participation of multiple receptors and the sophisticated process, it is commonly acknowledged that the main mechanism underlying lymphangiogenesis is through the VEGFR-3 signaling pathway (7), which enhances LEC survival, migration, and proliferation. After the interaction with high-affinity ligands VEGF-C and VEGF-D, VEGFR-3 is induced to form homodimers and VEGFR-2/-3 heterodimers, followed by phosphorylation and activation of the receptor (8). Cytoplasmic signaling mediators like Grb2 and SOS are then recruited to specific phosphorylated tyrosine sites to activate the subsequent Ras-Raf-MEK-ERK pathway. Concomitantly, other major downstream signaling, including PKC-dependent ERK, PI3K/Akt, and MKK4 mediated JNK1/2 pathways, are elicited *via* the corresponding phosphotyrosine residue sites (9). Multiple regulators such as membrane proteins β1 integrin (10), EphrinB2 (11), and co-receptor neuropilin 2 (Nrp2) (12), are proposed to facilitate receptor activation, internalization, or augment ligands’ affinity, respectively.

In addition, adjacent LECs interact with each other to promote lymphangiogenesis, mainly through Ang/Tie2, DLL4/Notch1, and EFNB2/EPHB4 signaling (13). The Ang/Tie pathway was newly detected for its extra function of facilitating tumor growth in a cervical cancer model (14), which drew increased attention for its potential as a novel target in treatments. Angiopoietin-1 causes autophosphorylation of the Tie-2 receptor, leading to FAK/ERK and PI3K/Akt stimulation. While PI3K/Akt plays a critical role in the process, it is stimulated by another high-yield mechanism, the HGF/HGFR signaling. There was increased c-MET expression observed in both inflammatory and tumor-induced lymphatic vessels, and HGF-c-MET interaction could indirectly upregulate the VEGF/VEGFR expression *via* activating NF-kB molecule (15). Furthermore, current research highlighted that the fatty acids β-oxidation participated in the LEC PROX-1 interaction with histone acetyltransferase p300 to enhance lymphangiogenesis (16). Other than those noted above, VEGF-A/VEGFR-2, EGFR, FGF, and PDGF were reported to have notable effects in lymphatic vessel remodeling as well, helping to define diverse potential anti-lymphangiogenic targets.

**Figure 1:**
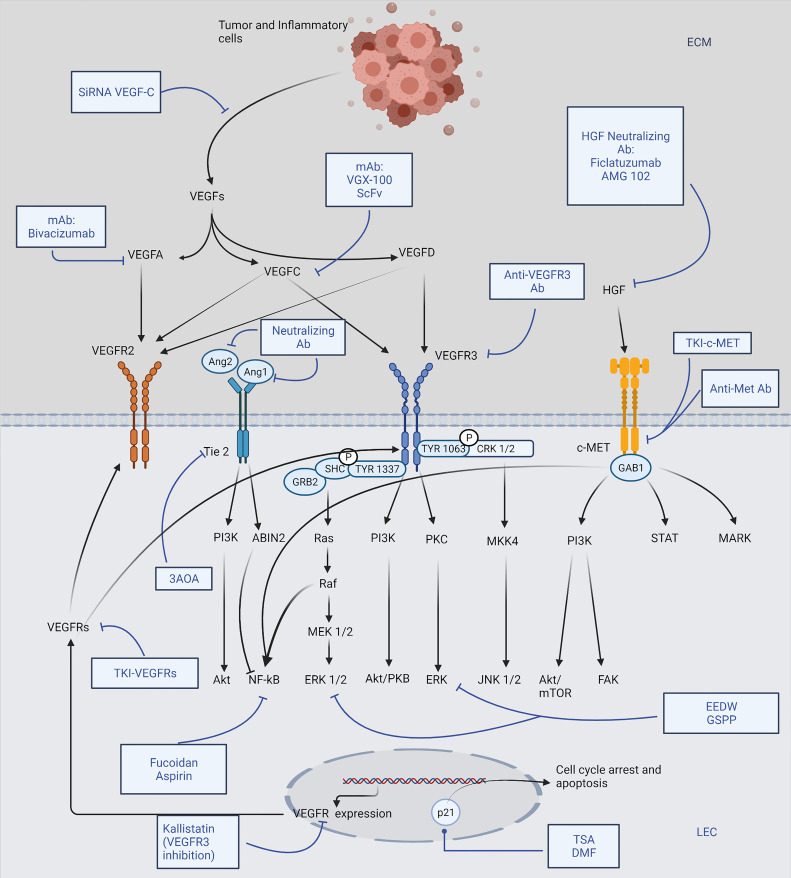
Brief scheme representing major lymphangiogenic pathways and drugs targeting lymphangiogenesis. Tumor cells and tumor-associated inflammatory cells induce LEC (lymphatic endothelial cell) proliferation, migration mainly through VEGF-C/-D expression, which activates subsequent VEGFR-2/-3 phosphorylation, leading to lymphangiogenesis. Various antibody drugs are shown to suppress the corresponding growth factors directly; membrane receptors, including VEGFRs, Tie2, and HGFRs, are also promising therapeutic targets. TKIs against VEGFRs and HGFRs work alongside the mAb (monoclonal antibody) and neutralizing antibodies; 3AOA (3-O-Acetyloleanolic acid) is a novel agent dampening Tie2 receptor. In addition, many drugs exert an inhibitory effect on the essential downstream pathways of the receptors: EEDW (Hedyotis diffusa Willd) and GSPP (Gekko Sulfated Glycopeptide) inhibit ERK signaling while Fucoidan and Aspirin effectively suppress NF-kB; TSA (Trichostatin A) and DMF (Dimethyl fumarate) could induce cell cycle arrest *via* upregulating p21 dependent pathways; Kallistatin and SiRNA VEGF-C are examples of drugs inhibiting VEGF-C and VEGFR-3 expression, respectively. The figure was created with BioRender.com.

Studies of lymphangiogenic pathway molecules have provided promising therapeutic targets and novel rationale for future cancer metastasis control (17) (**Figure 1**). So far, the main discovered molecules that serve as potential targets are VEGF-C, VEGF-D, VEGFR-2, VEGFR-3, HGF, and HGFRs. In earlier studies of the angiogenic process, many of these molecules have also shown their promoting effects on angiogenesis; and fortunately, until 2018, there have been 26 drugs approved by FDA for the anti-angiogenic therapy, with various indications (18). In recent years, during the concurrent studies that were carried out to detect approaches targeting lymphangiogenesis, multiple anti-angiogenic drugs exhibited inhibitory effects on lymphangiogenesis as well, advancing the progression in this field. According to their targets and modes of action, drugs targeting lymphangiogenesis can be categorized into several groups: 1) Antibody-based therapies (19), including monoclonal antibodies and some neutralizing antibodies or peptides directly targeting the VEGF-C/VEGFR-3 axis, which are currently tested in preclinical/clinical studies; the HGF/HGFR and newly identified Ang/Tie also served as their targets. 2) Small molecule kinase inhibitors, functioning as a separate group of drugs that efficiently dampen the common receptor pathways (20), among which several agents have already been approved for their anti-angiogenic effects and anti-tumor properties in clinical use; there are two major groups of receptor targets for the tyrosine kinase inhibitors, which are VEGFRs and HGFRs. 3) Preclinical candidate agents targeting the lymphangiogenic pathways at different levels via various mechanisms, including downregulation of the VEGF/VEGFR expression, induction of the p21 dependent pathway to trigger cell cycle arrest, and suppression of the Akt, ERK and NF-kB signaling.

## Drugs Targeting Tumor Lymphangiogenesis

### Antibody Drugs

Among the antibody drugs targeting the VEGF/VEGFR axis (**Table 1**), Bevacizumab was approved primarily as an anti-angiogenic drug in various malignancies, and more clinical trials were taken to assess its precise anti-lymphangiogenic effects in combination therapy. A current study showed that, by inhibiting VEGF-A induced inflammatory (lymph) angiogenesis, Bevacizumab could benefit corneal pathologies and was studied as a novel strategy in corneal and ocular surface diseases (41). In 2011, a human IgG subclass 1 mAb (monoclonal antibody) specific to VEGFR-3, IMC-3C5, entered the clinical trial and has recently completed the phase 1 study. It was shown to be well tolerated up to a dosage of 30 mg/kg weekly (qwk) in advanced solid tumors, though the following expanded evaluation of its activity on colorectal cancer (CRC) was minimal (22). Similarly, VGX-100 was another highly specific monoclonal antibody that explicitly binds to VEGF-C and dampens VEGFR-2 and VEGFR-3 activation. It entered the phase 1 trial of late-stage cancers, among which the primary indications were glioblastoma and metastatic CRC (42). VGX-100 was well tolerated when it was used alone or combined with Bevacizumab, and 12% of the evaluated patients obtained a best response of durable stable disease for more than four months (23). Another preclinical stage study revealed a phage-derived single-chain fragment of anti-VEGF-C mAb (anti-VEGF-C scFv). By interacting with the epitope on VEGF-C, scFv showed distinct specificity and affinity. Significant advantages of such drugs can be shown in cancers depending on the direct VEGF-C pathway for growth, like Kaposi sarcoma and acute myeloid or lymphocytic leukemia. Researchers also speculated additional benefits of these inhibitors in VEGF-C induced bone and macular degeneration (28). An IgG-like fusion protein molecule, VEGFR-31-Ig, which could simultaneously bind the angiogenic VEGF-A and the lymphangiogenic VEGF-C, has been reported. It was remarkable for its outstanding stability and comparable effect with the complex activity of VEGF-Trap (25 mg/kg) and sVEGFR-3 (25 mg/kg), whereas excluding their conventional drawbacks like impractical clinical use and inadequate preclinical safety (27). Also, based on a current database analysis of the VEGF-C/VEGFR-3 triggered KRAS/MAPK-YAP1/Slug signaling in skin cancer progression, a unique anti-VEGFR-3 peptide was discovered able to abrogate the process (25).

**Table 1:** Potential Ab drugs targeting lymphangiogenesis.

Target	Agent name	Mode of action	Indications	Status	Reference
**VEGF/VEGFR**	Bevacizumab	Anti-VEGF-A mAb	metastatic CRC, non-squamous non-small cell lung cancer, recurrent glioblastoma, hepatocellular carcinoma (HCC)	FDA-approved	(21)
	IMC-3C5 (LY3022856)	Anti-VEGFR-3 mAb	CRC	phase 1	(22)
	VGX-100	Anti-VEGF-C mAb	glioblastoma (GBM), metastatic colorectal cancers, prostate cancer	phase 1	(23)
	Diabody	Anti-VEGFR-2/VEGFR-3 mAb	ovarian cancer, prostate cancer, CRC	phase 1	(24)
	Anti-VEGFR-3 peptide	Anti-VEGFR-3 peptide	skin cancer	preclinical	(25)
	sVEGFR-3-Fc	Soluble VEGFR-3 decoy receptor	prostate cancer, melanoma	preclinical	(26)
	VEGFR-31-Ig	Receptor-Ig fusion protein (VEGF-A/VEGF-C)	HCC	preclinical	(27)
	single-chain fragment (scFv)	Anti-VEGF-C mAb fragment		preclinical	(28)
**HGF/c-MET**	Onartuzumab	Anti-MET Ab	non-small-cell lung cancer	phase 3	(29)
	ARGX-111	Anti-MET Ab	mammary carcinoma	phase 1	(30, 31)
	Telisotuzumab (ABT-700)	Anti-MET mAb	non-small-cell lung cancer (NSCLC)	phase 1	(32)
	Ficlatuzumab	HGF neutralizing Ab	head and neck squamous cell carcinoma; acute myeloid leukemia	phase 2	(33)
	Rilotumumab (AMG 102)	HGF neutralizing Ab	gastric cancer	phase 3	(34)
**Ang1/Ang2**	Trebananib (AMG 386)	Ang1/Ang2 neutralizing peptibody	recurrent ovarian cancer (phase 1/2 for various cancer types)	phase 3	(35)
	CVX-060	Ang2 neutralizing CovX-body	metastatic renal cell carcinoma	phase 1	(36)
	AMG 780	Ang1/Ang2 neutralizing Ab	advanced solid tumors	phase 1	(37)
	MEDI 3617	Ang2 neutralizing Ab	ovarian cancer	phase 1	(38)
	Nesvacumab/Aflibercept (REGN 910)	Ang2 neutralizing Ab	adrenocortical carcinoma, HCC	phase 1	(39)
	CVX-241	Ang2/VEGF neutralizing bispecific CovX-body	advanced solid tumors	phase 1	(40)

Another axis targeted by antibody drugs was the HGF/HGFR signaling. A phase 3 study of Rilotumumab (AMG 102) with Epirubicin, Cisplatin and Capecitabine (CX) as first-line therapy in advanced MET-positive gastric or gastroesophageal junction adenocarcinoma was taken. However, the trial was terminated with a disappointing result as the Rilotumumab group presented a lower median overall survival (OS) and 12-month survival rate compared to the placebo arm, and it was associated with frequent fetal events such as neutropenia (29%) and anemia (12%) (34). Filcatuzumab was assessed in combination with Cetuximab for refractory head and neck squamous cell carcinoma due to minor benefits shown by Cetuximab alone. The combination had an acceptable safety profile and a promising anti-tumor activity by dual inactivation of HGF and EGFR pathways, indicating an active mitigation of cancer progression (33). Nonetheless, results from a phase 3 study of Onartuzumab with Erlotinib in non-small cell lung cancer (NSCLC) were in stark contrast to the previous phase consequence, partially due to the small size of the phase 2 study (29). Although it is dubious that the selection of patients group would affect, future investigations were expected to pick splice-site mutations harbored tumor samples (43). Taken together with the failure to meet the endpoint by many other MET-signaling inhibitors, researchers implied an unsatisfactory perspective of Ab therapy targeting this axis.

Fortunately, several agents inhibiting the interaction between Angiopoietin-1/-2 and the Tie2 receptor were demonstrated with promising efficacy. Trebananib, an angiopoietin neutralizing peptibody, prolonged the progression-free survival in a phase 3 trial of patients with recurrent epithelial ovarian cancer, proving the effectiveness of targeting this non-VEGF pathway (35). But some recent trials of Trebananib in combination with standard ovarian cancer chemotherapy didn’t succeed in the overall survival improvement (44). Later designed AMG 780 had a relatively longer half-life duration (8 to 13 days) than that of Trebananib (3.1 to 6.3 days), and the phase 1 study of AMG 780 suggested a maximum dose of up to 30 mg/kg every 2 weeks in patients with advanced solid tumors (37). Selective anti-Ang2 mAb MEDI3617 was studied for its recommended monotherapy dosage and effects when combined with Bevacizumab and several other chemotherapies. Within the recommended single-agent dosage (1000 mg Q2W; 1500 mg Q3W), there was no worsening of adverse effects like edema in combined treatments, yet the study of MEDI 3617 monotherapy in advanced ovarian cancers was discontinued due to the observation of peripheral edema (38). More recently, Nesvacumab showed preliminary anti-tumor activity in a phase 1 trial and the safety profile was acceptable at all dose levels tested (39). Combination therapy of Nesvacumab with Aflibercept was also well tolerated (45). Some additional investigations were taken in retinal diseases like macular degeneration though they failed to meet the phase 2 endpoint (46). Some chemically programmed antibodies behaving as potent endothelin receptor antagonists, like CVX-060 and CVX-241, were designed to target Ang2. However, CVX-241 was prematurely terminated because of the absence of ideal pharmacological effects through the 25 mg/kg cohort and shorter than expected half-life duration (40). CVX-060 plus Axitinib were tested in patients with previously treated metastatic renal cell cancer, and as a result of the higher than anticipated treatment-related thromboembolic events, enrollment to the next part was discontinued, but alternate doses and/or disease settings were considered (36). Although peripheral edema occurred as common toxicity that appeared in anti-angiopoietin treatments, all the known cases were limited to grade 1 or 2, and no anti-VEGF therapy associated adverse effects were observed. Therefore, it might be noteworthy to combine anti-angiopoietin drugs with conventional inhibitors of VEGF pathways to further augment the overall survival in some refractory cases.

### Small Molecule Tyrosine Kinase Inhibitors

So far, FDA has approved several tyrosine kinase inhibitors (TKI) targeting VEGFRs for their significant improvement of progression-free survival in patients with renal cell carcinoma, thyroid cancer, hepatocellular carcinoma, and multiple other tumor types (**Table 2**). For example, in addition to the approved anti-angiogenic activity in malignancies, Pazopanib demonstrated favorable inhibitory effects on tumor growth, lymphatic metastasis, and tumor lymphangiogenesis in an orthotopic model of mice with colorectal cancer (56). Lenvatinib had versatile applications and predominantly suppressed lymph node metastasis in a breast cancer model, and for further studies of Lenvatinib-therapies, guiding biomarkers and sufficient selection of patients are needed (53). Triple angiokinase inhibitor, Nintedanib, adequately dampened lymphangiogenesis stimulated by VEGF-C, bFGF, and PDGF-BB in a suture-induced corneal neovascularization assay. Both topical and systemic applications showed positive results, though the *in vivo* safety of topical use wasn’t tested yet (52). Interestingly, other than the clinical application in renal cell carcinoma, Axitinib significantly inhibited LEC proliferation and lymphangiogenesis in a study of allergic asthma (68). An earlier establishment of Sunitinib in the breast cancer model presented marked reduction of tumor lymphatic and blood vessels density, as well as decreased axillary lymph nodes invasion, suggesting a prosperous blockade of LEC cellular function by dampening VEGFR-2/-3 activation from VEGF-C/-D (54). However, a controversy arose based on the observation of promoted lymphangiogenesis caused by the increase of VEGF-C transcription and mRNA stabilization in clear cell renal cell carcinomas (55). The paradoxical effects of Sunitinib treatment in different cancer models suggested a variation of LEC response in different tissues, implying a more appropriate patient sample will be needed for further confirmation of many other drugs also. It is noticeable that, as for future studies of these multi-kinase inhibitors, management of some common adverse effects, including drug-induced hypertension and dermatotoxicity like the hand-foot skin reaction, should be considered while estimating the clinical parameters.

**Table 2:** potential anti-lymphangiogenic TKIs targeting VEGFRs.

Target	Agent name	Indications	Status	Reference
**VEGFRs (PDGFR, c-Kit, MET, FLT3, RET)**	Cabozantinib	mast cell tumor, renal cell carcinoma	FDA-approved	(47, 48)
**VEGFRs (PDGFRs, FGFRs, BRAF, c-Kit, RET)**	Regorafenib	CRC, HCC, gastrointestinal stromal tumor	FDA-approved	(18, 49)
**VEGFRs (PDGFRs, c-kit, RET)**	Sorafenib	renal cell carcinoma, HCC, thyroid cancer	FDA-approved	(50, 51)
		desmoid tumors	phase 3	
**VEGFRs (PDGFRs, FGFR, PDGFR)**	Nintedanib	idiopathic pulmonary fibrosis, NSCLC	FDA-approved	(52)
		inflammatory corneal lymphangiogenesis	preclinical	
**VEGFR-2/-3**	Lenvatinib (MK-7902/E7080)	thyroid cancer	FDA-approved	(53)
		breast cancer	preclinical	
**VEGFRs (PDGFRs, c-kit, Flt3, RET)**	Sunitinib	renal cell carcinoma	FDA-approved	(54, 55)
		breast cancer	preclinical	
**VEGFRs (PDGFRs, c-kit)**	Pazopanib	renal cell carcinoma, soft tissue sarcoma	FDA-approved	(56)
		CRC	preclinical	
**VEGFRs (PDGFRs, c-kit)**	Cediranib	relapsed ovarian caner	phase 3	(57)
**VEGFRs (PDGFRs, FGFRs)**	Brivanib	HCC	phase 3	(58)
**VEGFRs (PDGFRs)**	SU-014813	metastatic breast cancer	phase 2	(59)
**VEGFR-3 (EGFR-1/-2/-4, Src family)**	JNJ-26483327	advanced solid tumors	phase 1	(60)
**VEGFR-2/-3**	L-783277 derivative 17	3D-microfluidic tumor lymphangiogenesis assay	preclinical	(61)
**VEGFR-3**	MAZ51	melanoma	preclinical	(62)
**VEGFR-3**	Toluquinol	corneal pathologies	preclinical	(63)
**VEGFR-2/-3 (ERK1/2, AKT)**	AD0157	breast cancer, human myeloid leukemia	preclinical	(64, 65)
**VEGFR-3 (more selective than VEGFR-1/-2)**	SAR 131675	diabetic nephropathy	preclinical	(66)
**VEGFR-3**	Ki23057	gastric carcinoma	preclinical	(67)

Concurrently, there are extensive ongoing trials and preclinical studies of novel therapeutics. Investigations have been taken to test the synergistic effect of combining Vatalanib (750 mg BID) with the mTOR inhibitor, Everolimus (10 mg QD), and a 20% increase in the response rate was detected through this combination (compared to Everolimus therapy alone) in neuroendocrine tumors (69). Selective dual inhibitor Brivanib was studied in patients with hepatocellular carcinoma who were intolerant to Sorafenib or for whom Sorafenib failed. Still, the trial didn’t meet the primary endpoint of overall survival, and similar negative results were taken by several other phase 3 trials of hepatocellular carcinoma (HCC) (58). Favorable results were shown in combination therapy of Cediranib and Olaparib (PARP inhibitor) with significantly extended effectiveness than Olaparib alone in patients having relapsed, platinum-sensitive ovarian cancer (57), which was consistent with previous studies. Moreover, preclinical studies of MAZ51 (a selective VEGFR-3 inhibitor) established a valid blockade of prostate tumor growth (70) and melanoma metastasis (62), as well as a 30% reduction in gastric cancer cell migration (71). Another highly selective strategy using SAR131675 has presented diminished lipotoxicity-induced lymphangiogenesis in a type 2 diabetic nephropathy model (66). AD0157 (a marine fungal metabolite), as a natural anti-angiogenic compound, was shown to possess a pro-apoptotic property in human myeloid leukemia cells by inducing the caspase-dependent cascades (64). Concomitantly, other than the caspase-dependent apoptosis, it could also attenuate tumor-associated lymphangiogenesis and metastatic dissemination to both regional lymph nodes and distant organs by decreasing VEGFR-3/-2, ERK1/2, and Akt phosphorylation in human myeloid leukemia cells (65).

The other potent group of TKI is the MET kinase inhibitor (**Table 3**). In the recent two years, FDA granted approval to selective MET inhibitor Capmatinib and Tepotinib for adult patients with metastatic NSCLC whose tumors were MET exon 14 mutated. However, adverse effects like phototoxicity and interstitial lung diseases were reported (74). In the study of EMD 1214063 and EMD 1204831, both candidates triggered tumor regression in a dose-related fashion by inhibiting c-MET phosphorylation, regardless of HGF-dependent or HGF-independent activation. Comparatively, the former was more sustained. A single dose of EMD 1214063 at 10 or 30mg/kg could achieve a complete and persistent target inhibition, making it better than many other MET inhibitors (81). PF-04217903 is one of the most selective c-MET inhibitors. It provided a robust TGI (tumor growth inhibition) in the MET-amplified GTL-16 model and a U87MG model exhibiting the HGF/c-MET autocrine loop (80). In clinical trials, multi-kinase inhibitor Crizotinib was approved for the treatment of ALK-positive NSCLC. It was relatively non-toxic though resistance may occur within one year (72). Several other drugs were discontinued in the study, like AMG 337, which was terminated early because of low response (13% of evaluable patients) observed during the phase 2 trial partially due to the larger sample and narrower range of tumor types (77).

**Table 3:** potential anti-lymphangiogenic TKIs targeting HGFRs(c-MET).

Target	Agent name	Indications	Status	Reference
**c-MET (ALK, ROS1)**	Crizotinib	sarcoma and sarcomatoid malignancies harboring ALK fusions	FDA-approved	(72, 73)
**c-MET**	Capmatinib	NSCLC	FDA-approved	(74)
**c-MET**	Tepotinib (MSC 2156119/EMD 1214063	NSCLC	FDA-approved	(74)
**c-MET**	Tivantinib	NSCLC, HCC	phase 3	(75)
**c-MET**	Savolitinib	NSCLC	phase 2	(76)
**c-MET**	AMG 337	gastric/gastric esophageal junction/esophageal tumors	phase 2	(77)
**c-MET/Ron**	AMG 208	prostate cancer	phase 2	(78)
**c-MET (VEGFR-2)**	Foretinib	glioblastoma, gastric cancer	phase 2	(79)
**c-MET**	PF-04217903	CRC	phase 1	(80)
**c-MET**	EMD 1204831	solid tumors	phase 1	(81)
**c-MET/Ron**	MK-8033	advanced cancer	phase 1	(82)

### Candidate Agents

A previous study utilizing the CI66 tumor model suggested that the siRNA-mediated VEGF-C gene silencing could efficiently suppress tumor lymphangiogenesis as well as recruitment of inflammatory cells in TME (tumor microenvironment) and calcium carbonate nanoparticle was believed to be a prosperous vector for it (**Table 4**). Furthermore, a higher level of dendritic cells and concanavalin A-induced proliferation in tumor-associated leukocytes was associated with the siVEGF-C application, indicating an additional positive modulation of the immune response (95). *In vitro* study of Fucoxanthin showed decreased lymph node metastases and significant reduction of *in vivo* LVD (lymphatic vessel density) in an MDA-MB-231 breast cancer model. Enhanced blocking of matrix metalloproteinases (MMP) secretion and increased TIMP-1 protein expression were also demonstrated in the experimental data of Fucoxanthin (84). Shikonin, according to the report, possessing a favorable inhibitory effect of NF-kB nuclear translocation and activation, causing the decrement of LEC cord formation (87). As an endogenous angiogenic inhibitor, Kallistatin was shown to be a potential dual-effect substance disrupting both VEGFR-2 & VEGFR-3. Because of severe adverse effects appearing in VEGFR TKIs treatment, combination therapy of Kallistatin with the kinase inhibitors could thus be considered a new strategy (85). Meanwhile, a highly metastatic human lung adenocarcinoma cell line Anip973 revealed an increase in both VEGF-C & COX-2 immunoreactivity, indicating a possible correlation in between. Therefore COX-2 (largely from PGE2-stimulated EP4 receptors) and EP4 may serve as novel targets for treatment in light of its potential regulatory activity on VEGF-C expression (83). Many other agents like Fucoidan (89), Aspirin (90), Hedyotis diffusa Willd (EEHDW) (91), and Curcumin (93) were able to block the important downstream signalings of VEGFR-3 in various cancer models. Interestingly, an earlier study explicated an advantageous inhibitory effect of VEGF-A induced lymphangiogenesis and sentinel lymph node metastasis in oral cancer by 3AOA. Besides the VEGF-A inhibition, 3-O-Acetyloleanolic acid (3AOA) also interfered with an alternative lymphangiogenic pathway mediated by Ang-1/Tie-2 in the CT-26 colon carcinoma model. 5 μM 3AOA could significantly dampen 87% of human umbilical vein endothelial cells (HUVEC) proliferation, migration, and tube formation stimulated by Ang-1 (96, 97). Similarly, 6,8-Diprenylgenistein (6,8-DG), an isoflavonoid isolated from Cudrania tricuspidata has been reported with its inhibitory effect on VEGF-A induced lymphangiogenesis as well. In the oral cancer model, 6,8-DG inhibited VEGF-A expression and blocked the VEGFR-2 interaction with VEGF-A, decreasing cervical lymph nodes metastasis of the oral squamous cell carcinoma (101). Newly examined histone deacetylase inhibitors Trichostatin A (TSA) (100) and Dimethyl fumarate (DMF) (94) both caused G1 cell cycle arrest in a p21-dependent manner. DMF had a pronounced anti-tumorigenic effect for melanoma and a cell type-specific cell cycle arrest. No apoptotic influence was observed in DMF-treated human DLEC (dermal lymphatic endothelial cells), while TSA, on the other hand, downregulated the anti-apoptotic proteins cIAP-1/2, causing cell death.

**Table 4:** Preclinical anti-lymphangiogenic candidate agents.

Agent name	Mode of action	Conventional usages	Novel indications	Reference
**Celecoxib**	COX-2 inhibition	osteoarthritis, rheumatoid arthritis	highly metastatic lung adenocarcinoma	(83)
**Fucoxanthin**	VEGF-C/VEGFR-3 depression; NF-kB degradation	obesity, diabetes mellitus	breast cancer	(84)
**Kallistatin**	inhibition of VEGFR-3 expression	vascular and organ injury	gastric cancer	(85)
**Oxyresveratrol**	downregulation of VEGF-C/VEGFR-3 expression	hyperpigmentation disorders	HCC	(86)
**Shikonin**	inhibition of NF-kB/HIF-1α pathway	flat wart, psoriasis		(87)
**Qingjie Fuzheng Granules(QFG)**	suppression of VEGF-C/VEGFR-3 dependent PI3K/Akt pathway	cancer (as adjuvant therapy)	CRC	(88)
**Fucoidan**	inhibition of NF-kB/PI3K/Akt pathway	dietary supplements		(89)
**Aspirin**	inhibition of NF-kB/VCAM-1 pathway	angina pectoris, ankylosing spondylitis		(90)
**EEHDW (Hedyotis diffusa Willd**	inhibition of ERK, PI3K/Akt, STAT3 pathway	cancer (as adjuvant therapy)	CRC	(91)
**GSPP (Gekko Sulfated Glycopeptide)**	inhibition of bFGF induced ERK1/2 signaling	cancer (as adjuvant therapy)		(92)
**Curcumin**	inhibition of HMGB1/VEGF-C, VEGFR-2/-3 signaling	dietary supplement, food additive	gastric cancer	(93)
**Dimethyl fumarate**	induction of G1 cell cycle arrest	multiple sclerosis, psoriasis		(94)
**SiRNA VEGF-C**	downregulation of VEGF-C expression		breast cancer	(95)
**3AOA (3-O-Acetyloleanolic acid)**	inhibition of Ang-1/Tie‐2; suppression of VEGF-A induced VEGFR-1/-2 phosphorylation		colon cancer, oral squamous cell carcinoma	(96, 97)
**Phomaketide A**	inhibition of VEGFR-3, PKCδ, and eNOS signaling cascades			(98)
**LHbisD4 (heparin conjugate)**	blockade of VEGF-C induced signaling pathway		breast cancer	(99)
**Trichostatin A**	induction of G0/G1-arrest			(100)

## Discussion

Tumor-induced lymphangiogenesis and therapeutics providing virtual management of lymphatic metastasis will continue their upward trend in advances based on the successive achievements. Targeting the VEGF-C/VEGFR-3 at different levels remained the major approach due to the increasing confirmation for its essential role in the lymphangiogenic mechanism and a growing understanding of the axis. Newly discovered targets still need further investigation for their accurate regulatory effects in various tumors, whereas some of them, like angiopoietin and genetic transcription factors, were already considered plausible in addition to the VEGF/VEGFR pathway to reduce the common adverse effects associated with VEGF therapies. Although there haven’t been any FDA-approved anti-lymphangiogenic drugs yet, accumulated favorable results, especially from small molecular VEGFR inhibitors, were presented in clinical studies. Most of the examined TKIs were selected from existing anti-angiogenic applications and exhibited promising therapeutic effects. However, single-agent treatment, for example, with monoclonal antibodies alone, received minor therapeutic responses; thus, current anti-lymphangiogenic investigations focus more or less on combining anti-lymphangiogenic methods with other standard anti-tumor drugs due to the extended involvement of different mechanisms in cancer progression. It is noteworthy that, apart from the medications from anti-angiogenic therapy, many preclinical studies (**Table 4**) have discovered some conventional drugs and molecules with a more predominant effect on lymphangiogenesis; simultaneously, applications with a selective VEGF-C and VEGFR-3 depression were designed. In order to develop more drugs with specific anti-lymphangiogenic effects and find out the best strategy in each case to delineate the differences between anti-angiogenic and anti-lymphangiogenic therapy, several traditional Chinese herbs with conventional anti-tumor effects also enrolled in recent studies. Interestingly, they exhibited effective anti-lymphangiogenic activity *via* different mechanisms, shedding a light on a new group of potential therapeutics in this field.

Since angiogenesis, lymphangiogenesis, and neoplasia share many similar mechanisms and pathways, it’s still necessary to continue to uncover some basic problems, such as what factors keep the blood vasculature apart from the lymphatic vessels after embryonic differentiation and the functional differences of each factor in blood endothelial cells and lymphatic endothelial cells. Also, some recent research pointed out that the LEC genetic pattern strongly influenced the lymphovascular response to the factors in different tissues, causing both functional and structural heterogeneity of tumor lymphangiogenesis. Hence, revealing specific molecular biomarkers in each particular cancer and tracing tumor LEC genetic lineage for their precise roles and mechanisms might be the upcoming challenges in future studies. Moreover, according to the paradoxical effects of lymphatic vessels in immune-cell trafficking, an accurate balance between the protumor and anti-tumor immune response is needed in order to improve the benefits of anti-lymphangiogenic therapies.

## Author Contributions

Literature review and writing—original draft preparation: CW. Writing—review and editing: MC. Supervision and funding acquisition: MC. All authors have read and agreed to the published version of the manuscript.

## Funding

This work was supported by National Natural Science Foundation of China (81603119) and Natural Science Foundation of Beijing Municipality (7174316).

## Conflict of Interest

The authors declare that the research was conducted in the absence of any commercial or financial relationships that could be construed as a potential conflict of interest.

## Publisher’s Note

All claims expressed in this article are solely those of the authors and do not necessarily represent those of their affiliated organizations, or those of the publisher, the editors and the reviewers. Any product that may be evaluated in this article, or claim that may be made by its manufacturer, is not guaranteed or endorsed by the publisher.
